# An Oleanolic Acid Derivative Inhibits Hemagglutinin-Mediated Entry of Influenza A Virus

**DOI:** 10.3390/v12020225

**Published:** 2020-02-18

**Authors:** Mengdie Ye, Yixian Liao, Li Wu, Wenbao Qi, Namrta Choudhry, Yahong Liu, Weisan Chen, Gaopeng Song, Jianxin Chen

**Affiliations:** 1 Guangdong Provincial Key Laboratory of Veterinary Pharmaceutics Development and Safety Evaluation, Guangzhou 510642, China; mengdieye@stu.scau.edu.cn (M.Y.); choudharynamrta@gmail.com (N.C.); gale@scau.edu.cn (Y.L.); 2 College of Veterinary Medicine, South China Agricultural University, Guangzhou 510642, China; 13503033623@139.com (L.W.); qiwenbao@scau.edu.cn (W.Q.); 3 College of Materials and Energy, South China Agricultural University, Guangzhou 510642, China; cjjwcf@163.com; 4 Department of Biochemistry and Genetics, La Trobe Institute for Molecular Science, La Trobe University, Melbourne, Victoria 3086, Australia; weisan.chen@latrobe.edu.au

**Keywords:** oleanane-type derivatives, influenza A virus (IAV), virus entry inhibitors, hemagglutinin (HA)

## Abstract

Influenza A viruses (IAV) have been a major public health threat worldwide, and options for antiviral therapy become increasingly limited with the emergence of drug-resisting virus strains. New and effective anti-IAV drugs, especially for highly pathogenic influenza, with different modes of action, are urgently needed. The influenza virus glycoprotein hemagglutinin (HA) plays critical roles in the early stage of virus infection, including receptor binding and membrane fusion, making it a potential target for the development of anti-influenza drugs. In this study, we show that OA-10, a newly synthesized triterpene out of 11 oleanane-type derivatives, exhibited significant antiviral activity against four different subtypes of IAV (H1N1, H5N1, H9N2 and H3N2) replications in A549 cell cultures with EC_50_ ranging from 6.7 to 19.6 μM and a negligible cytotoxicity (CC_50_ > 640 μM). It inhibited acid-induced hemolysis in a dose-dependent manner, with an IC_50_ of 26 µM, and had a weak inhibition on the adsorption of H5 HA to chicken erythrocytes at higher concentrations (≥40 µM). Surface plasmon resonance (SPR) analysis showed that OA-10 interacted with HA in a dose-dependent manner with the equilibrium dissociation constants (KD) of the interaction of 2.98 × 10^−12^ M. Computer-aided molecular docking analysis suggested that OA-10 might bind to the cavity in HA stem region which is known to undergo significant rearrangement during membrane fusion. Our results demonstrate that OA-10 inhibits H5N1 IAV replication mainly by blocking the conformational changes of HA2 subunit required for virus fusion with endosomal membrane. These findings suggest that OA-10 could serve as a lead for further development of novel virus entry inhibitors to prevent and treat IAV infections.

## 1. Introduction

Influenza A virus (IAV), the causative agent of influenza, is a major pathogen that causes public health problem and socioeconomic burden world-wide. With waterfowl as the primary reservoir, the virus is able to infect a wide variety of birds and mammals, including humans. Due to this trait, zoonotic spillovers occur occasionally and can lead to pandemics with severe consequences for the human population [1,2]. The avian-origin H5N1 and H7N9 subtypes of influenza viruses are recent examples of animal viruses that acquired the potential to infect and cause disease in humans. H5N1 IAV are often highly pathogenic (HP) avian influenza viruses. In 1997 in Hongkong, six people died out of 18 confirmed human cases with HP H5N1 virus infection [3]. In 2003, novel H5N1 IAV genetic variants circulated in Southeast Asian countries, which led innumerable poultry to death and caused sporadic human infections in the following years. By the end of 2017, 860 laboratory confirmed cases of H5N1 IAV infection from 16 different countries, resulting in 454 deaths, had been reported to the World Health Organization (WHO) [2]. Influenza A viruses mutate easily because of their segmented RNA genome, making it difficult to produce a timely and sufficiently effective vaccine to prevent the potential epidemic outbreaks. Therefore, using anti-influenza agents could be a more efficient approach for prevention and treatment at the beginning of outbreak. To date, two types of anti-influenza drugs have been approved. One type pertains to the M2 ion channel inhibitors, including amantadine and rimantadine, which block the release of viral RNA into the cytoplasm [4]. The others are neuraminidase (NA) inhibitors, including zanamivir, oseltamivir and peramivir, which prevent progeny virus from being released by their host cells [5,6]. However, it has been reported that most of the circulating IAV strains are resistant to M2 inhibitors and may rapidly develop resistance to NA inhibitors, which limits the use of those licensed drugs [7,8,9]. Therefore, it is urgently required to develop novel anti-influenza agents preferentially with novel mechanisms of action to combat influenza, especially the HP influenza.

The first and most critical step of IAV infection is viral entry mediated by the interaction of viral envelope protein hemagglutinin (HA) and its receptor on host cell surface [10]. HA forms a trimer including three HA1 and three HA2 subunits. Each HA2 contains a fusion peptide, a soluble ectodomain (SE) and a transmembrane domain [11]. After binding to receptor through HA1, IAV is endocytosed. Within the endosome, increasing acidity, pH 5–6, induces HA2 to undergo an irreversible conformational change, leading to the fusion peptide extruding toward the endosomal membrane, and ultimately fusion of the viral and endosomal membranes [10]. HA2, as a major component of the stem region of HA, is a highly conserved subunit. Consequently, blocking HA2′s conformational change could abolish viral membrane fusion and infection. Hence, HA2 is considered an attractive antiviral target [12,13]. The first discovered compound of this class was tertiary butylhydroquinone [14]. Later, several compounds that act in a similar way were described: BMY-27709, CL-385319, stachyflin and 4c [15,16,17,18]. However, these small molecular compounds were reported to display inhibitory effects on H1, H2 and H3 IAV subtypes rather than H5 subtype. Recently, guided by structural knowledge on the interactions of HA and anti-stem broadly neutralizing antibodies, Maria and colleagues successfully developed a small molecule JNJ4796 with the ability to inhibit HA-mediated fusion and protect mice against lethal and sublethal influenza challenges [19]. The potent anti-IAV activity of JNJ4796 further reinforces that HA2 could be a promising drug target for blocking IAV infection.

Pentacyclic triterpenoids (PTs), an abundant natural product in plants, have received considerable attention due to their wide spectrum of antiviral activities against various viruses, such as HIV, IAV and HCV [20,21,22,23], and some are already marketed as therapeutic agents or dietary supplements. For example, glycyrrhizin (glycyrrhizic acid) has been used for treating chronic hepatic diseases for over 40 years in China and Japan clinically.

Oleanane acid (OA) triterpene is probably the most well-known member of PTs with noticeable antiviral activities. Echinocystic acid (EA), an oleanane-type triterpene, was reported to have substantial inhibitory activity on HCV entry with EC_50_ at nanomolar range [24,25]. Zhou reported that a different chemical modification to the C-17-COOH of OA led to Y3 (an OA-acetyl galactose conjugate), which showed strong antiviral activity against H1N1 and H3N2 IAV infections in vitro [13]. Further study indicated that Y3 was able to bind tightly to HA protein, thereby disrupting the interaction of HA with its sialic acid receptor [13]. In our previous study, we used an efficient HIV-based pseudotyping system to screen a semisynthetic saponin library, and discovered that OA saponins with *β*-chacotriosyl residue at the C-3-OH of OA exhibited excellent inhibitory activity against H5N1 IAV entry [26]. Based on this finding, we further designed, synthesized and evaluated a series of 3-O-*β*-chacotriosyl oleanolic acid analogs as H5N1 IAV entry inhibitors. We found that the introduction of a disubstituted amide structure at the 17-COOH of OA could significantly improve the selective index while maintaining their antiviral activities in vitro [27]. However, as the previous antiviral evaluation of 3-O-*β*-chacotriosyl oleanolic acid analogs was based on a pseudotyping system, their inhibitory effects on IAV and mechanisms of action are unconfirmed. In the present study, antiviral activities of OA and its eleven analogs, including four newly synthesized derivatives, against H5N1 virus in A549 cells, were investigated, and their mechanisms of action and antiviral activities against other IAV subtypes based on a representative novel compound OA-10 were further investigated. To our knowledge, this is the first report on anti H5N1 IAV activity of OA derivatives.

## 2. Materials and Methods

### 2.1. Compounds

**Figure 1:**
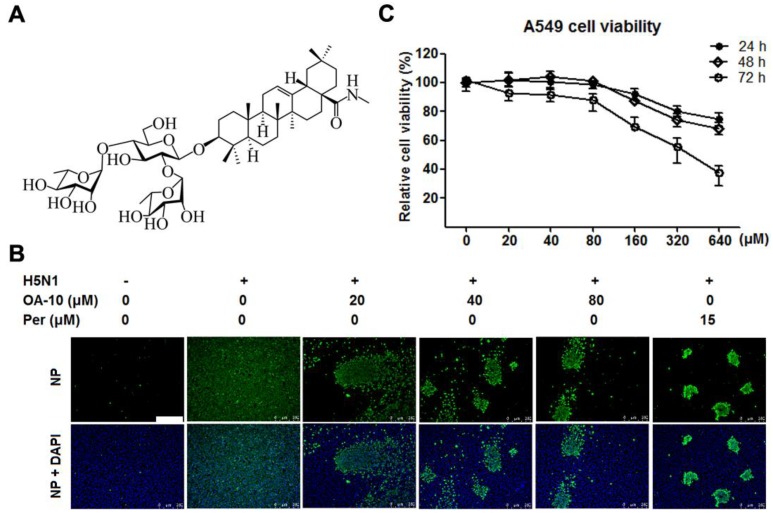
OA-10 inhibited H5N1 IAV replication in A549 cells with minimal cytotoxicity. (**A**) Chemical structure of OA-10. (**B**) Antiviral activity of OA-10 against H5N1 IAV infection. Confluent A549 cells grown in 96-well plates were infected with H5N1 IAV (0.1 MOI) for 1 h at 37 °C and then cultured in fresh medium containing various concentrations of OA-10 or 15 µM peramivir (Per). At 24 hpi, the cells were fixed with paraformaldehyde and the viral NP expression was detected by indirect immunofluorescence assay (IFA). Representative IFA images from three independent experiments are shown. Scale bar: 250 µm. (**C**) Cytotoxicity of OA-10 in A549 cells. The results are expressed as percentages (%) of mock treated cells.

In total, 12 oleanane-type triterpenoid compounds (Figure S1 and Figure 1A), including oleanolic acid and its 11 derivatives, and their antiviral activities against H5N1 IAV infection in A549 cells were studied. Oleanolic acid was purchased from Chengdu Gelipu Biotechnology Co., Ltd. (Chengdu, China). Seven derivatives named OA-1, OA-2, OA-3, OA-4, OA-5, OA-6 and OA-7 were synthesized as described previously [27]. Four new derivatives named OA-8, OA-9, OA-10 and OA-11, which were confirmed by data from nuclear magnetic resonance (NMR) and high-resolution electrospray ionization mass spectrometry (HRESIMS) analysis (shown in the supplementary material), were synthesized by a method similar to that described previously [27]. Briefly, the known saponin 3-(*β*-d-glucopyranosyl)-oleanolic acid benzyl ester or 3-(*α*-d-mannopyranosyl)-oleanolic acid benzyl ester was subjected to PivCl in the presence of pyridine to selectively protect the 3,6-OHs of the *β*-glucopyranosyl or *α*-mannopyranosyl residue, and subsequent glycosylation of the 2, 4-OHs in sugars with 2,3,4-tri-*O*-acetyl-L-rhamnopyranosyl trichloroacetimidate using TMSOTf as the promoter, followed by deprotection of the acyl groups with NaOH to produce two tridesmosidic oleanolic acid benzyl ester saponins, respectively. Hydrogenolysis of the above oleanolic acid benzyl ester saponins over palladium/carbon in THF-MeOH led to OA-9 and 3-O-*β*-chacotriosyl oleanolic acid, was carried out to furnish OA-10 or OA-11 by four consecutive acetylation, acyl chlorination, amide condensation reaction and alkaline hydrolysis, respectively. The purities of all oleanane-type triterpenoids were ≥98% by HPLC analysis. Ribavirin hydrochloride was purchased from Guangdong Starlake Bioscience Co. Ltd. (Zhaoqing, China) with purities ≥ 99%. Peramivir in sterile 0.9% NaCl solution (0.3 g/100 mL) was purchased from Guangzhou Nucien Pharmaceutical Co., Ltd. (Guanzhou, China). Arbidol hydrochloride was purchased from Dalian Melun Biological Technology Co., Ltd. (Dalian, China) with purities ≥98%. Oleanane-type triterpenoids were dissolved in dimethyl sulfoxide (DMSO) and diluted with PBS to <0.4% DMSO for in vitro experiments. Ribavirin hydrochloride was dissolved in PBS for in vitro experiments.

### 2.2. Cell Lines and Influenza Virus

A549 (human lung carcinoma) cells and MDCK (Madin-Darby canine kidney) cells used for IAV infection were cultured in Dulbecco’s modified Eagle’s medium (DMEM, Gibco, UT, USA) supplemented with 10% fetal bovine serum (FBS, Biological Industries, Kibbutz Beit Haemek, Israel), 100 U/mL of penicillin and 100 µg/mL streptomycin in a humidified atmosphere with 5% CO_2_. All cells were obtained from the Center of Cellular Resource, Chinese Academy of Sciences (Shanghai, China).

Avian influenza A virus strain A/Duck/Guangdong/99(H5N1 IAV) and A/Chicken/Guangdong/16/ 1996 (H9N2) were kindly provided by the Veterinary Technology Center of South China Agricultural University (Guangzhou, China). The H1N1 IAV strain A/Puerto Rico/8/34 (PR8) and H3N2 IAV strain A/Guangdong/Dongguan/1100/2006 viruses were obtained from the Chinese Center for Disease Control and Prevention (Beijing, China). Virus stocks were passaged in 10-day old embryonated chicken eggs for 48 h. The allantoid fluid was harvested and aliquots were stored at −80 °C until required. Experiments involving H5N1 virus were conducted in a physical containment level three (PC3) laboratory.

### 2.3. Cytotoxicity Assay

The cytotoxicity of tested compounds was evaluated using a MTT assay as described previously by Luo [28]. Briefly, cells were grown in 96-well plates for 24 h. The medium was replaced with fresh one containing serially diluted compounds and the cells were further incubated for 24 h, 48 h or 72 h. The culture medium was removed and replaced with 100 µL 3-(4,5-dimethylthiozol-2-yl)-3,5-dipheryl tetrazolium bromide (MTT; Sigma-Aldrich, MA, USA) solution (1 mg/mL in PBS) and incubated at 37 °C for 4 h. After removal of the supernatant, 150 µL of DMSO was added to all of the wells to dissolve the formazan crystals for 10 min at 37 °C. Cell viability was then measured as the absorbance at 490 nm with a microplate reader (Thermo fisher scientific, MA, USA) and expressed as a percentage of the control level. The mean optical density (OD) values from six replicated wells per treatment were used as the cell viability index. The 50% cytotoxic concentration (CC_50_) was calculated by GraphPad Prism 7.0 (GraphPad Software, San Diego, CA, USA).

### 2.4. Indirect Immunofluorescence Assay (IFA)

Indirect immunofluorescence assay was used to rapidly evaluate antiviral activities of compounds against H5N1 IAV infection. For immunostaining, the H5N1-infected or control cells were fixed with 4% paraformaldehyde for 10 min, then permeabilized with 0.25% Triton X-100 for 10 min at room temperature (RT). Cells were blocked with 1% bovine serum albumin (BSA) for 60 min at RT and then incubated with a mouse monoclonal antibody against IAV nucleoprotein (NP protein) (1:500 dilution, Sino Biological, Beijing, China) at 4 °C overnight. After three washes with PBS, the cells were incubated for 1 h at RT with an anti-mouse IgG antibody conjugated with Alexa Fluor^®^ 488 (green) (Cell Signaling Technology, MA, USA) at 1:1000 dilution. Nuclei were counterstained with 50 µL of 4,6-diamidino-2-phenylindole (DAPI, 300 nM; Sigma-Aldrich, MA, USA). Immunofluorescence was captured using the Leica DMI 4000B fluorescence microscope (Leica, Wetzlar, Germany). Blue and green fluorescence spots were counted as the total and IAV-infected cell numbers respectively in every IFA image.

Relative NP protein level (%) of each image was calculated based on the fluorescence optical density (OD) using Software Image J. Results from compound-treated samples were compared to those from corresponding DMSO-treated control groups (set as 100%). The EC_50_ value (the concentration required to protect 50% cells from IAV infection) was determined by plotting the relative NP protein level as a function of compound concentration and calculated using GraphPad Prism 7.0 software (GraphPad Software, San Diego, CA, USA).

### 2.5. In Vitro Virus Growth Inhibition Assay

A549 monolayers were infected with the virus for 1 h. Supernatants were removed, and cells were then incubated with DMEM containing serial concentrations of test compound. Cells and supernatants were collected at indicated time points post-infection and in total subjected to three freeze-thaw cycles at −80 °C and 4 °C to ensure maximal release of cellular virions [29]. Final viral titers in the supernatants were determined by an end point dilution assay using MDCK cells and expressed as log_10_TCID_50_ /0.1 mL.

### 2.6. Real-Time Reverse-Transcription PCR (RT-PCR)

Total RNA was extracted from cells or mixtures of cells and supernatants using the total RNA rapid extraction kit (Fastagen, Shanghai, China) following the manufacturer’s instructions. RNA was reverse-transcribed into first-strand cDNA using a reverse transcription kit (TaKaRa, Japan). PCR amplification was performed using 1 μL of reverse-transcribed product with primers designed for IAV-NP and GAPDH (glyceraldehyde-3-phosphate dehydrogenase, used as the endogenous control). The primers used for PCR amplification are listed in Table 1 [30]. Real-time PCR was performed using 2×RealStar Green Power Mixture (containing SYBR Green I Dye) (Genstar, Beijing, China) on a CFX96 Real-time PCR system (Bio-Rad, Hercules, CA, USA). Relative mRNA expression was calculated by 2^−ΔΔCT^ method using DMSO-treated infected cells as reference samples for determining IAV-NP [31,32]. To assess the effect of OA-10 on transcriptional activation of NP in IAV infected cells, the relative fold change of each NP gene expression was calculated and compared between OA-10-treated virus-infected and virus-infected cells.

### 2.7. Time Course Inhibition Assay

To estimate the influence of OA-10 on the IAV replication cycle, A549 cells were grown in 24-well plates to confluence and then infected with H5N1 IAV (0.1 MOI) for 1 h at 37 °C. OA-10 or ribavirin or peramivir was added before, during or after IAV infection. For pretreatment, cells were incubated with the indicated compound for 2 h at 37 °C, followed by three washes with PBS and then infected with H5N1 IAV for 1 h. For co-treatment, cells were simultaneously incubated with H5N1 IAV and the compound. After 1 h, the virus–drug mixture was removed, and the cells were washed three times with PBS before fresh medium was added. For post-treatment, cells were first infected with H5N1 IAV for 1 h followed by three washes with PBS and then incubated in the fresh medium containing the compound. At 24 hpi, progeny viruses in the supernatants were determined by an end point dilution assay and the extent of virus infection in the cells was assessed by IFA for NP protein, respectively.

To determine the specific stage(s) of the viral life cycle affected by OA-10, a time-of-addition assay was performed as described by Luo et al. [28]. Briefly, confluent monolayers of A549 cells grown in 24-well plates were infected with H5N1 IAV (1.0 MOI) for 1 h at 37 °C. Cells were washed three times with PBS to remove unbound viruses and incubated in fresh medium. In total, 80 µM of OA-10 or ribavirin or peramivir was then added for 0 to 2, 2 to 4, 4 to 6, 6 to 8 h or 0 to 8 hpi. After each incubation period, the cells were washed three times with PBS and incubated with fresh medium at 37 °C. At 8 hpi, the cells were subjected to viral NP protein analysis using IFA and viral mRNA analysis using RT-PCR, respectively.

### 2.8. Hemagglutination Inhibition Assay (HAI)

The inhibitory activity of OA-10 on HA-mediated hemagglutination of chicken red blood cells (RBCs) was assessed by an HAI assay. Briefly, 25 µL of A/Duck/Guangdong/99(H5N1) (4 hemagglutination units) was incubated with 25 µL H5 standard antiserum (H5 antiserum were provided by the Veterinary Technology Center of South China Agricultural University, Guangzhou, China) or OA-10 at indicated concentrations for 30 min at RT. Then, 50 µL chicken RBCs (0.5%) in saline solution were added to each well and incubated at 37 °C for 30 min. The plates were taken images at 45 degree inclination for recording hemagglutination and the fluidity of deposited RBCs.

### 2.9. Hemolysis Inhibition Assay (HIA)

The inhibitory effects of compounds on virus-induced hemolysis at low pH were determined by a procedure described previously by Basu et al. [33]. Briefly, chicken RBCs were washed twice with PBS and resuspended to 2% (vol/vol) in PBS and stored at 4 °C until use; 100 µL of compound diluted in PBS was then mixed with an equal volume of H5N1 IAV (10^8^ PFU/mL) in a 96-well plate. After incubating the virus-compound mixture at room temperature for 30 min, 200 µL of 2% chicken RBCs (pre-warmed at 37 °C) was added. The mixture was incubated at 37 °C for another 30 min. To trigger hemolysis, 100 µL of sodium acetate-acetic acid buffer solution (0.5 M, pH 5.2) was added and mixed with the RBC suspension. The mixture was incubated at 37 °C for another 30 min for HA acidification and hemolysis. To separate unlysed RBC from the lysed ones, plates were centrifuged at the end of incubation at 1200 rpm for 6 min; 300 µL of the supernatant was transferred to another flat-bottom 96-well plate. The OD_540_ was read on a microtiter plate reader. IC_50_ is defined as the compound concentration that generates 50% protection on chicken RBCs lysed by virus.

### 2.10. Surface Plasmon Resonance (SPR) Analysis

Interactions between the influenza HA and the compounds were analyzed using the Berthold bScreen LB 991 (LabXMedia group, Midland, Canada) at 4 °C. Recombinant influenza HA protein (Abcam, Cambridge, England) from virus strain A/Vietnam/1203/2004 (H5N1) was immobilized on a sensor chip (Photo-cross-linker SensorCHIP™) using an amine coupling kit (GE Healthcare, Buckinghamshire, UK). Subsequently, compounds were injected as analytes at various concentrations, and we used PBST (10 mM phosphate buffer with 0.1% Tween 20, pH 5.0) as running buffer. For binding studies, analytes were applied at corresponding concentrations in running buffer at a flow rate of 30 μL/min with a contact time of 600 s and a dissociation time of 360 s. Chip platforms were regenerated with regeneration buffer (Glycine-HCl, pH = 2.0) after each test cycle. The processing and analyses of association and dissociation rate constants (Ka/Kon and Kd/Koff respectively) and the equilibrium dissociation constant (KD, kd/ka) were performed using the data analysis software of the bScreen LB 991 unlabelled microarray system according to a single-site binding model (1:1 Langmuir binding) with mass transfer limitations for binding kinetics determination.

### 2.11. Molecular Docking

The protein structure of hemagglutinin (PDB ID: 6CFG) was used for the docking study. All calculations were performed using Discovery Studio 2017. The 3D structures of OA-10 were constructed using the Discovery Studio small molecule window, and energy was minimized by CHARMm force field in a two-step method: steepest descent with RMS gradient convergent to 0.1, and the final step was conjugate gradient with RMS gradient convergent to 0.0001. Prior to the docking procedure, all bound water molecules were removed from the protein crystal structure. A site sphere radius was set to 13.39 Å in which the other parameters were set as default. The docking program CDOCKER was used to perform the automated molecular simulation in which the top hits were set as 10, and the random conformations were set as 20. The top compounds were ranked by the corresponding values of -CDOCKER interaction energy.

### 2.12. Statistical Analysis

All values are expressed as means ± SDs from at least three independent experiments. Statistical significance was determined by Student’s *t*-test when only two groups were compared or by one-way analysis of variance (ANOVA) when more than two groups were compared. Statistical analyses were performed using GraphPad Prism 7 (GraphPad Software, San Diego, CA, USA). * *P* < 0.05, ** *P* < 0.01 and *** *P* < 0.001 were considered to be statistically significant at different levels.

## 3. Results

### 3.1. Compound OA-10 Inhibits IAV Infections in A549 Cells with Minimal Cytotoxicity

Chemical structures of oleanane acid (OA-0) and its 11 derivatives, including four new derivatives, are shown in Figure S1 of the Supplementary Material. The cytotoxicity of OA-0 and its derivatives on A549 cells was first evaluated using the 3-(4,5-dimethylthiozol-2-yl)-3,5-dipheryl tetrazolium bromide (MTT) assay. For each compound, CC_50_ value, the concentration required to reduce normal cell viability by 50% after 24 h of compound treatment, was determined, as shown in Table 2. Derivatives OA-1, OA-2, OA-4, OA-5, OA-7, OA-8 and OA-9 exhibited greater cytotoxicity on A549 cells with CC_50_ ≤ 20.5 µM, while other derivatives and oleanolic acid exhibited less cytotoxicity with CC_50_ ≥ 31.1 µM. Noticeably, OA-10 showed the least cytotoxicity with CC_50_ > 640 µM (Figure 1C). DMSO (0.4%, used as solvent) did not exhibit detectable cytotoxicity on A549 cells**.** No obvious cytotoxicity was observed for OA-10 at concentrations ≤ 80 µM after 24, 48 or 72 h of treatment, as shown in Figure 1C. Thus, 80 µM of OA-10 was selected as the maximum concentration for further studies.

The antiviral activities of synthesized OA derivatives against H5N1 IAV were evaluated via relative nucleoprotein (NP) expression level (%) detected by immunofluorescence microscopy at 24 hpi. The initial results showed that OA-0 and the 11 derivatives exhibited various antiviral activities against H5N1 IAV infection (Table 2). It was found that OA-0 and all other derivatives except OA-6 significantly reduced H5N1 IAV infection in A549 cells with EC_50_ ≤ 14.2 µM, while OA-6 showed the lowest antiviral activity with EC_50_ > 86.6 µM. However, most of the effective derivatives, including OA-1, OA-2, OA-3, OA-4, OA-5, OA-7, OA-8, OA-9 and OA-11, exhibited high cytotoxicity on A549 cells with CC_50_ ≤ 31.1 µM, while OA-0, OA-10 and OA-11 showed relatively lower cytotoxicity on A549 cells with CC_50_ ≥ 60.0 µM. Most interestingly, OA-10 showed significant inhibition on H5N1 IAV replication in a dose-dependent manner in concentrations ranging from 20 to 80 µM (EC_50_: 14.0 µM) with very low cytotoxicity, as shown in Figure 1B,C. Among the 12 evaluated compounds, OA-10 showed the highest select index (SI > 45). Thus, OA-10 was selected for further studies.

To explore whether OA-10 possesses a broad inhibitory effect on different IAV subtypes, three other IAV strains (PR8, H9N2 and H3N2) were evaluated using IFA and A549 cells at 24 hpi. As shown in Figure S2A and Table 3, OA-10 also exhibited significant inhibitions on PR8 (EC_50_: 6.7μM), H9N2 (EC_50_: 15.3 μM) and H3N2 (EC_50_: 19.6 μM) replications.

**Figure 2:**
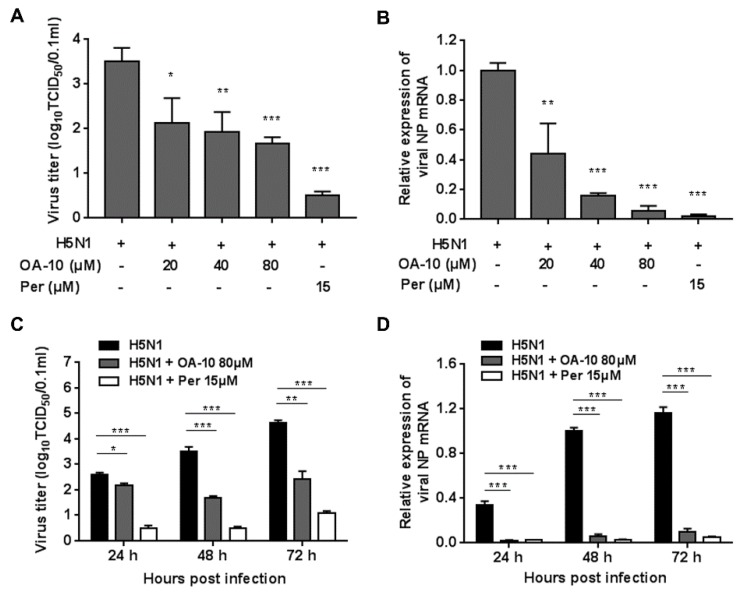
OA-10 inhibited H5N1 IAV infection in A549 cells. A549 cells grown in 24-well plates were infected with H5N1 IAV (0.1 MOI) for 1 h, and then cultured in fresh medium containing various concentrations of OA-10 or 15 µM peramivir. At 48 hpi (**A**,**B**) or indicated time-points post infection (**C**,**D**), cells and supernatants of each well were mixed and subjected to viral titer or RT-PCR analysis. H5N1 IAV expression of GAPDH was used as a loading control, and a DMSO-treated sample (H5N1 IAV infected and non-drug treated) at 48 hpi was used as treatment control (set as 1). Data are presented as means ± SDs of results from three independent experiments. *P < 0.05, ** *P* < 0.01 and *** *P* < 0.001 compared to the respective virus control.

To more accurately assess OA-10′s inhibitory role, we further examined its antiviral effects against H5N1 IAV infection using virus titration and RT-PCR at 48 hpi. As shown in Figure 2A, treatment with OA-10 resulted in a significant reduction of progeny virus titer in a dose-dependent manner. Treatment with 80 μM of OA-10 led to a 1.8 log reduction in progeny virus production compared to that in DMSO-treated control. In fact, OA-10 at concentrations from 20 to 80 μM significantly inhibited H5N1 IAV NP RNA levels in A549 cells in a dose-dependent manner (Figure 2B). We further studied the viral inhibition kinetics by OA-10 at 80 μM. In the H5N1 IAV-infected control, the levels of virus titer and virus mRNA expression increased continuously from 24 to 72 hpi (Figure 2C,D). The addition of 80 μM OA-10 significantly reduced progeny virus titers and viral RNA levels at all time-points, as shown in Figure 2C,D. Simultaneously, treatment with OA-10 also reduced progeny virus titer on PR8, H9N2 and H3N2 infection, respectively, in a dose-dependent manner at 48 hpi, as shown in Figure S2B. Peramivir, a well-known neuraminidase inhibitor, was used as a positive antiviral drug control in this study. Our results showed that 15 μM of peramivir exhibited a significant inhibition on IAV infections in the same assays.

### 3.2. OA-10 Interferes with Virus Entry

**Figure 3:**
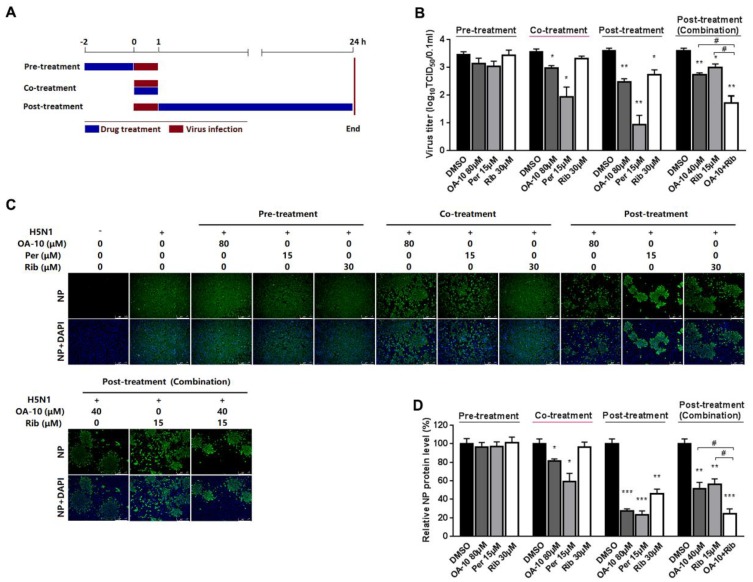
OA-10 exhibited inhibition on H5N1 IAV replication in co- and post-treatment modes. A549 cells grown in 24-well plates were treated with indicated compounds for 2 h prior to virus infection (pre-treatment), or for 1 h during viral infection (co-treatment), or for 24 h after 1 h virus infection and removal (post-treatment) (**A**). For three treatment models, 0.1 MOI of H5N1 IAV was used for infecting cells for 1 h. At 24 hpi, supernatants were collected for determining virus titer using the end point dilution assay (**B**), and the cells were subjected to viral NP protein analysis using IFA (**C**,**D**). Results shown in (**D**) are normalized NP protein levels based on the fluorescence optical densities (OD) of the images from three independent experiments. Software Image J was used to digitize image OD. Results from OA-10 or peramivir or ribavirin treated samples were compared to those from corresponding DMSO-treated control groups (set as 100%) (**D**). Representative IFA images of the three independent experiments are shown in C. Scale bar: 250 µm. * *P* < 0.05, ** *P* < 0.01 and *** *P* < 0.001 compared to the respective virus (DMSO-treated) control.

To identify the OA-10 affected stage(s) during an influenza infection cycle, we first performed a virus binding (attachment) assay by coculture A549 cells with IAV at 4 °C to permit attachment yet avoid viral entry in the presence or absence of OA-10. The co-incubation of 80 μM OA-10 with H5N1 IAV at 4 °C for 1 h followed by removal of excess virus and 37 °C culture did not affect IAV infection (Figure S3), indicating that OA-10 does not affect IVA binding to cells and also does not directly inactivate IAV particles. We next performed time course studies for the inhibitory effects of OA-10. A549 cells were treated with OA-10 for 2 h prior to virus infection (pre-treatment), or for 1 h during the viral infection (co-treatment), or for 24 h after 1 h virus infection and removal (post-treatment), as shown in Figure 3A. Results in Figure 3B–D show that pre-treatment of 80 μM OA-10 did not reduce progeny virus yield and viral NP production. This result indicates that OA-10 does not impair the susceptibility of A549 cells to IAV infection. When cells were incubated with H5N1 IAV in the presence of 80 μM OA-10 for 1 h (co-treatment), a mild but significant reduction in progeny virus yield as well as NP protein expression was observed. This result indicates that OA-10 likely interacts with the virus early during the infection cycle. When cells were treated with 80 μM OA-10 for 24 h post H5N1 IAV infection (post-treatment), one log reduction in progeny virus yield was observed (Figure 3B), which was also reflected by decreased NP production (Figure 3C,D). This result is consistent with the above results and further indicates that OA-10 likely exerts its antiviral effects during virus entry. In these assays, 15 μM peramivir exhibited more significant inhibition on H5N1 IAV’s replication in both co-treatment and post-treatment modes, while 30 μM ribavirin exhibited inhibition only in the post-treatment mode, and its inhibition was significantly weaker than that of peramivir. In addition, the combination of 40 μM OA-10 and 15 μM ribavirin treatment showed a synergistic effect on reducing progeny virus titer and NP production, suggesting their complimentary inhibiting mechanisms against IAV replication (Figure 3B–D).

**Figure 4:**
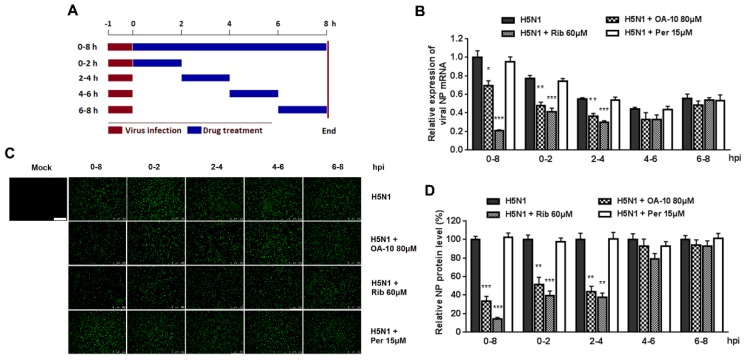
OA-10 inhibits H5N1 IAV replication by targeting the earlier stage(s) of the viral infection cycle. A549 cells grown in 24-well plates were infected with 1.0 MOI of H5N1 IAV for 1 h. After two washes with PBS, OA-10 or ribavirin or peramivir was added at the indicated time points and removed after 2 h or 8 h. After each incubation period, the cells were incubated in fresh medium, harvested at 8 hpi and NP mRNA and protein analyzed by RT-PCR and IFA, respectively. (**A**) The experimental design of time-of-addition assay. (**B**) Relative virus NP mRNA level. (**C**,**D**) Viral NP protein expression, representative IFA images (**C**) and digitized NP expression (**D**) from three independent experiments. Scale bar: 250 µm. * *P* < 0.05, ** *P* < 0.01 and *** *P* < 0.001 compared to the respective virus control.

It was estimated that from IAV entering into a cell to producing its progeny takes on average 6–8 h, depending on cell type [34]. To identify the exact stage(s) affected by OA-10 during such a IAV replication cycle, we treated the infected cells with OA-10 at four separate 2 h time intervals (0–2, 2–4, 4–6 or 6–8, and 0–8 h as a control) and monitored viral NP RNA and protein expression (Figure 4A). As shown in Figure 4B, a more significant inhibition of viral replication, as represented by decreased viral NP RNA level, was observed when OA-10 was added to the A549 cells during 0–2 and 2–4 hpi. A similar profile was also observed in viral NP expression (Figure 4C,D). These results again indicate that OA-10 exerts its effect during the early stages of IAV infection; i.e., virus internalization, endosome fusion, RNA release and replication. Ribavirin inhibits viral RNA synthesis. As expected, it exerted better IAV inhibition when added to the infected A549 cells before 4 hpi and showed no inhibition when added after 4 hpi. On the other hand, peramivir blocks progeny virus release from infected cells, and did not exhibit any inhibition of either viral RNA synthesis or NP protein expression during a single infection cycle in this assay, although its antiviral activity was the most remarkable, as shown in Figure 3, when multiple infection cycles were studied.

### 3.3. OA-10 Partly Blocks IAV HA Adsorption to Chicken RBCs at High Concentrations

**Figure 5:**
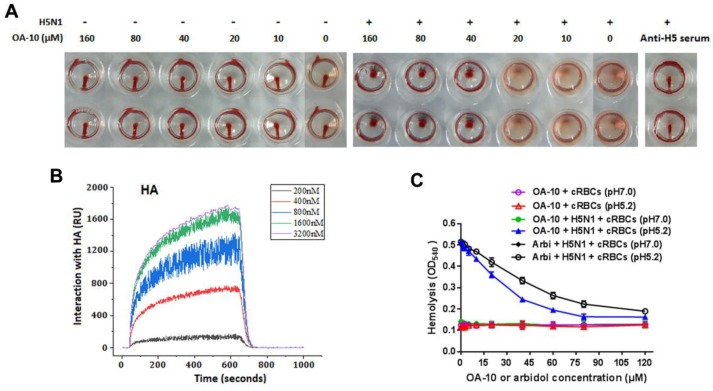
HA is a potential target of OA-10. (**A**) Comparisons of the behaviors of OA-10 vs. antisera against H5 HA in inhibition of H5N1 IAV-induced aggregation of chicken RBCs. OA-10 partially blocked H5N1 virus adsorption to chicken RBCs at concentrations ≥40 µM. (**B**) Characterization of the affinity between OA-10 and HA protein using surface plasmon resonance (SPR) analysis. (**C**) Inhibition of OA-10 on HA-mediated chicken RBCs hemolysis using arbidol as the positive control.

IAV is able to adsorb to chicken RBC, resulting in hemagglutination through the interaction of the receptor-binding domain in viral HA1 subunits with the sialic acid receptors on RBC membrane. To see whether HA is the potential target of OA-10, we questioned whether OA-10 could inhibit hemagglutination by interfering with H5 HA adsorption to RBC. OA-10 did not inhibit HA adsorption to chicken RBCs at concentrations of 10 and 20 µM. At the concentrations of OA-10 ≥ 40 µM, it was observed that a portion of RBCs settled to the bottom of the wells but the settled RBCs were not able to flow, as shown in Figure 5A. Meanwhile, OA-10 treatment did not affect RBC properties in the absence of H5 virus, and the positive control antisera against H5 hemagglutinin (Anti-H5) could effectively inhibit H5 HA adsorption to chicken RBCs with a titer at 1:16, and the settled RBCs could move when the plate was tilted (Figure 5A). These results indicated that OA-10 might have a weak interaction with the receptor binding domain of H5 HA1, which needs further demonstration.

### 3.4. OA-10 Exhibits a Strong Interaction with HA Protein

To investigate the interaction of OA-10 with HA, we studied the affinities of OA-10 with HA by surface plasmon resonance (SPR). A recombinant HA protein from virus strain A/Vietnam/1203/2004 (H5N1) was used as representative HA of H5N1 IAV strains, because the amino acid sequence identity of the full-length HA proteins between A/Vietnam/1203/2004 strain and A/Duck/Guangdong/99(H5N1) strain used in above antiviral assay was 97.0% (Supplementary Material 5) [35]. As shown in Figure 5B, OA-10 interacted with HA in a dose-dependent manner, and the equilibrium dissociation constant (KD) of the interaction was 2.98 × 10^−12^ M, indicating a strong affinity between OA-10 with HA. OA-10 did not interact with negative control protein FKBP12 (FK506 binding protein 1A, 12 kDa), indicating that interactions of HA with OA-10 were specific [36]. This result indicates that HA is most likely the target of OA-10 by which it inhibits virus entry and subsequent replication.

### 3.5. OA-10 Inhibits H5N1 IAV-Mediated Hemolysis at Low pH

We next investigated whether OA-10 was able to inhibit HA-induced chicken RBC hemolysis at a low pH [33]. To trigger hemolysis, the virus-cell suspension was acidified (pH 5.2) to initiate HA subunit HA2 conformational changes that lead to the lysis of chicken RBCs and their hemoglobin release. Arbidol, a licensed antiviral agent against IAV infection in Russia and China, was demonstrated to inhibit IAV replication by binding to HA2 in acidic condition and used as a positive hemolysis inhibitor control in this study [37]. As shown in Figure 5C, as expected, arbidol showed hemolysis inhibition with an IC_50_ of 51 µM. Interestingly, OA-10 more potently inhibited HA-induced hemolysis in a dose-dependent manner, with an IC_50_ of 26 µM. Taken together, these results show that OA-10 interacts with HA2.

### 3.6. Molecular Model of HA-OA-10 Binding

**Figure 6:**
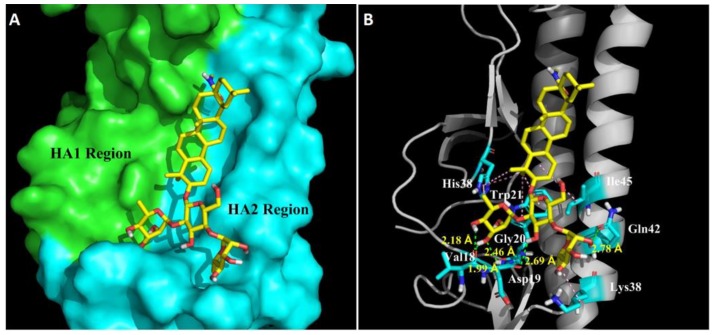
Structural model of OA-10 binding site in H5 HA. (**A**) CR6261 antibody binding site surface. The modeling was based on the published crystal structure of the A/Vietnam/1203/2004 (H5N1) (PDB: 6CFG). HA1 and HA2 regions are highlighted in green and blue, respectively. Compound OA-10 is shown as yellow sticks. (**B**) Modeled structure of HA complexed with OA-10. Green lines represent hydrogen bond interaction, with the distances indicated; and pink lines represent hydrophobic interaction between OA-10 and HA.

In order to further understand the molecular basis of OA-10 interacting with HA, we performed docking experiments using CDOCKER protocol to predict the binding modes of OA-10 into the CR6261 antibody binding site which is a highly conserved hydrophobic groove at the HA1-HA2 interface in the HA stem region [19]. The docked conformation of OA-10 was determined based on the minimum CDOCKER interaction energy. As shown in Figure 6, the modeled structure of HA complexed with OA-10 indicated that C2-OH and C3-OH of the L-rhamnose moiety linked to C2-OH of D-glucose were positioned within appropriate distance (2.46 Å, 1.99 Å and 2.18 Å) to make three strong hydrogen bonds with Gly20 and Val18 in HA2, respectively. Furthermore, C3-OH of D-glucose and the C2-OH of the L-rhamnose moiety linked to C4-OH of D-glucose formed hydrogen bonds with Asp19 and Gln42 with the distances 2.69 Å and 2.78 Å, respectively. In addition, oleanane scaffold also form numerous hydrophobic contacts with the HA1 residue (His38) and HA2 residues (Trp21, Lys38 and Ile45). According to docked conformation of OA-10, 3-O-*β*-chacotriosyl moiety obviously plays an important role in binding residues in HA2 with higher docking score.

## 4. Discussion

In the present study, OA-10, a newly synthesized oleanane-type triterpene, exhibits significant antiviral activity against highly pathogenic H5N1 IAV replication with an EC_50_ of 14.0 μM in A549 cell cultures. The in vitro cytotoxicity of OA-10 is quite low, with a CC_50_ of more than 640 μM and a selective index of more than 45. OA-10 also exhibits similar inhibitory effect on other IAV subtypes including PR8, H3N2 and H9N2, with EC_50_ values ranging from 6.7 to 19.6 μM. The time course inhibition study indicates that OA-10 exerts its antiviral effect during early stages of viral infection post binding to cell surface receptors. SPR analyses demonstrate that OA-10 interacts with HA protein strongly. The inhibition of OA-10 on H5N1 IAV induced hemolysis of chicken RBCs at low pH confirms interaction of OA-10 with HA subunit HA2. Furthermore, computer-aided molecular docking analysis suggested that OA-10 might bind to the interface of HA1 and HA2 in the HA stem region, which was known to undergo significant rearrangement during membrane fusion [19].

One IAV replication cycle is orderly composed of virus binding, internalization, RNA replication and viral protein synthesis, assembly, budding and release from infected cells [38]. Through the time course inhibition experiments, we found that OA-10 inhibited IAV replication in the co- and post-treatment modes (Figure 3), but was unable to block IAV binding to A549 cells (Figure S3). To identify the exact stage(s) of IAV replication cycle affected by OA-10, we investigated the time course inhibition within one IAV lifecycle and found that OA-10 exerted its effect during the early stages of IAV infection (Figure 4). During this stage, IAV attachment to target cells is mediated by HA1 via sialic acid-receptor binding, and subsequent virus-endosome membrane fusion is mediated by rearrangement of HA2 at low pH. Our results showed that OA-10 did not block IAV binding to cells at 4 °C (Figure S3). In addition, OA-10 did not inhibit IAV adsorption to chicken RBCs at concentrations of 10 and 20 µM, in spite of the fact that non-classical hemagglutination inhibitions of OA-10 at 40 and 80 µM were observed. These results suggest that the HA1 receptor binding domain is likely not a main target. Given that OA-10 exhibited antiviral activity during early stage(s) of IAV infection cycle, we speculated that OA-10 might target the membrane fusion step mediated by the more conserved hemagglutinin transmembrane subunit HA2. This hypothesis was confirmed by the activity of blocking hemolysis of OA-10 in a low pH environment in a dose-dependent manner (Figure 5C), as hemolysis is mediated by HA2 rather than HA1 [39].

To investigate the binding intensity of OA-10 with HA, SPR analyses were conducted. SPR data showed that OA-10 interacted with HA strongly with KD of 2.98 × 10^−12^ M (Figure 5B), which was consistent with its potent hemolysis inhibition at low pH. To study the possible binding site, docking simulation analyses were performed, by which a highly conserved hydrophobic cavity at the HA1-HA2 interface in the HA stem region was identified as the possible binding site of OA-10. The binding cavity is formed by residues of the two HA subunits, including Gly20, Val18, Trp21, Lys38 and Ile45 in HA2, and His38 in HA1, which was previously reported to be one of the critical regions responsible for the conformational changes in HA2 at low pH [19]. In addition, this region is recognized by an antibody with a broad-spectrum neutralizing ability to avian and human influenza A viruses [19]; thus, it could serve as a potential drug target for developing IAV entry inhibitors. Indeed, our data indicated that OA-10 might bind to this cavity through hydrogen bonds and hydrophobic interaction.

Influenza A viruses have been classified into 18 hemagglutinin subtypes (H1 to H18), which can be divided phylogenetically into two groups (1 and 2). H1, H5 and H9 belong to Group 1 HA of IAV, while H3 belongs to Group 2 HA of IAV [40]. It is known that the amino acid sequence identities of the HA2 portion between different HA subtypes are much higher than those of the full-length HA proteins [40], which makes HA2 an attractive target for developing broad-spectrum therapeutic antibodies and antiviral drugs. In fact, several broadly neutralizing antibodies (bnAbs) against IAV conserved HA stem have been developed, and their broad protection against IAV infection has been demonstrated in clinical trials. One such examples is the antibody CR6261 for most group 1 IAV subtypes [41,42]. Recently, guided by structural knowledge on the interactions of HA and anti-stem bnAb CR6261, a small molecule JNJ4796 that mimics the bnAb functionality with the ability to inhibit HA-mediated fusion was successfully developed. Importantly, this compound demonstrated potent antiviral activities against H1 and H5 strains in vitro and in vivo [19], but not group 2 IAVs such as H3 and H7 subtypes [19]. An OA derivative Y3 was reported to have significant antiviral activity in vitro against H3 and H1, suggesting broad-spectrum inhibitions of OA derivatives against both Group 1 and Group 2 IAVs.[13]. Further, HR2 in influenza HA2 was recently shown to be the target domain for Y3 [43]. In the present study, we show that OA-10 has promising antiviral activities against four IAV subtypes, including H1N1, H5N1, H9N2 (Group 1 IAVs) and H3N2 (Group 2 IAV), further demonstrating the potential of OA derivatives as broad-spectrum IAV entry inhibitors. To our knowledge, this is the first reported antiviral activity of OA derivatives against H5N1 IAV infection. Further in vivo studies will be carried out to clarify the efficacy of OA-10 as an IAV entry inhibitor in animals.

Another potential advantage of OA-10′s antiviral activity is its synergistic inhibition on viral replication when used together with ribavirin, a broad-spectrum virus RNA polymerase inhibitor (Figure 3). Such synergy was likely the result of the simultaneous disruption of HA-mediated viral entry by OA-10 (earlier step) and polymerase-mediated RNA replication by ribavirin (later step). It is expected that using an inhibitor like OA-10 together with ribavirin or NA inhibitors would not only enhance antiviral effects against IAV infection, but also prevent or significantly delay drug resistance.

In summary, we demonstrate that OA-10, a novel synthesized oleanane-type triterpenoid, inhibits four different IAV subtypes, including Group 1 H1N1 (PR8), H5N1 and H9N2, and Group 2 H3N2 infections with potent activity and negligible cytotoxicity in A549 cells. Mechanically, OA-10 blocks the conformational changes of the HA2 subunit at the low pH required for IAV to fuse with an endosomal membrane. These effects are attributed to a conserved hydrophobic cavity in the HA stem region as the likely binding site of OA-10. It could serve as a lead for optimization in order to design novel compounds with improved antiviral potency. OA-10 and its derivatives hold promise to be developed as broad-spectrum anti-influenza drugs.

## Figures and Tables

**Table 1:** Real-time PCR primer sequences.

Name ^a^	Sequences 5′ to 3′
NP-F	5′-GGATTTGGCGTCAAGCGAACA-3′ - 3′ACCAGAAGATKTGTCMTTCCAGGG -3′
NP-R	5′-GTCCCTACCCCCTTTACTGC-3′ TACTCCTCCGCATTGTCTCCGAAG -3′
GAPDH-F	5′-GCACCGTCAAGGCTGAGAAC-3′
GAPDH-R	5′-TGGTGAAGACGCCAGTGGA-3′

^a^ F: forward primer; R: reverse primer

**Table 2:** Cellular toxicity and inhibitory activity of oleanane-type triterpenoid derivatives against H5N1 influenza A virus (IAV) replication in A549 cells.

Compound	MW (g/mol)	CC_50_^a^ (μM)	EC_50_^b^ (μM)	SI ^c^
OA-0	456	67.4 ± 4.8	14.2 ± 1.9	4.7
OA-1	911	15.1 ± 1.3	2.30 ± 0.4	6.6
OA-2	996	20.5 ± 1.2	5.22 ± 0.6	3.9
OA-3	939	31.1 ± 2.5	2.87 ± 0.4	11
OA-4	966	12.1 ± 1.0	4.45 ± 0.7	16
OA-5	897	7.55 ± 0.8	4.24 ± 0.6	1.8
OA-6	924	>640	>86.6	-
OA-7	924	9.88 ± 1.1	7.79 ± 1.1	1.3
OA-8	924	15.5 ± 1.5	3.03 ± 0.5	5.1
OA-9	911	10.8 ± 0.9	4.61 ± 0.8	2.3
OA-10	924	>640	14.0 ± 2.3	>45
OA-11	1000	60.0 ± 4.5	6.7 ± 0.9	9.0

^a^ CC_50_, the concentration required to reduce normal, noninfected cell viability by 50%; ^b^ EC_50_, the concentration required to protect 50% cells from H5N1 IAV infection; ^c^ SI (selectivity index) is the ratio of CC_50_ to EC_50_. Data were presented as means ± SDs of results from three independent experiments.

**Table 3:** Inhibitory activity of OA-10 against influenza A virus replication in A549 cells.

IAV Strain	H5N1	PR8 (H1N1)	H9N2	H3N2
EC_50_ (μM)	14.0 ± 2.3	6.7 ± 1.4	15.3 ± 2.5	19.6 ± 3.7

Data are presented as means ± SDs from three independent experiments.

## Supplementary Materials

The following are available online at https://www.mdpi.com/1999-4915/12/2/225/s1: Figure S1: Chemical structures of oleanane acid (OA-0) and its 11 derivatives. OA-8, OA-9, OA-10 and OA-11 were newly synthesized derivatives. Figure S2: OA-10 inhibits IAV PR8 (H1N1), H9N2 and H3N2 replications in A549 cells. Figure S3: OA-10 does not block IAV binding to A549 cells. Figure S4: Amino acid sequence alignment of the full-length HA proteins between strains of A/Vietnam/1203/2004 (H5N1) and A/Duck/Guangdong/99(H5N1).

## References

[1] Yen H.-L., Webster R.G. (2009). Pandemic influenza as a current threat. Curr. Top. Microbiol. Immunol..

[2] World Health Organization Influenza (Avian and Other Zoonotic). https://www.who.int/en/news-room/fact-sheets/detail/influenza-(avian-and-other-zoonotic).

[3] Bender C., Hall H., Huang J., Klimov A., Cox N., Hay A., Gregory V., Cameron K., Lim W., Subbarao K. (1999). Characterization of the surface proteins of influenza A (H5N1) viruses isolated from humans in 1997-1998. Virology.

[4] Hsieh H.-P., Hsu J.T.A. (2007). Strategies of development of antiviral agents directed against influenza virus replication. Curr. Pharm. Des..

[5] Xie Y., Xu D., Huang B., Ma X., Qi W., Shi F., Liu X., Zhang Y., Xu W. (2014). Discovery of N-Substituted Oseltamivir Derivatives as Potent and Selective Inhibitors of H5N1 Influenza Neuraminidase. J. Med. Chem..

[6] Kelly H., Cowling B.J. (2015). Influenza: The rational use of oseltamivir. Lancet.

[7] Gupta R.K., Nguyen-Van-Tam J.S. (2006). Oseltamivir resistance in influenza A (H5N1) infection. New Engl. J. Med..

[8] Le Q.M., Kiso M., Someya K., Sakai Y.T., Nguyen T.H., Nguyen K.H.L., Pham N.D., Ngyen H.H., Yamada S., Muramoto Y. (2005). Avian flu—Isolation of drug-resistant H5N1 virus. Nature.

[9] Moscona A. (2009). Global Transmission of Oseltamivir-Resistant Influenza. New Engl. J. Med..

[10] Liu S., Li R., Zhang R., Chan C.C.S., Xi B., Zhu Z., Yang J., Poon V.K.M., Zhou J., Chen M. (2011). CL-385319 inhibits H5N1 avian influenza A virus infection by blocking viral entry. Eur. J. Pharmacol..

[11] Ranaweera A., Ratnayake P.U., Weliky D.P. (2018). The Stabilities of the Soluble Ectodomain and Fusion Peptide Hairpins of the Influenza Virus Hemagglutinin Subunit II Protein Are Positively Correlated with Membrane Fusion. Biochemistry.

[12] Vanderlinden E., Naesens L. (2014). Emerging Antiviral Strategies to Interfere with Influenza Virus Entry. Med. Res. Rev..

[13] Yu M., Si L., Wang Y., Wu Y., Yu F., Jiao P., Shi Y., Wang H., Xiao S., Fu G. (2014). Discovery of pentacyclic triterpenoids as potential entry inhibitors of influenza viruses. J. Med. Chem..

[14] Bodian D.L., Yamasaki R.B., Buswell R.L., Stearns J.F., White J.M., Kuntz I.D. (1993). Inhibition of the fusion-inducing conformational change of influenza hemagglutinin by benzoquinones and hydroquinones. Biochemistry.

[15] Luo G., Torri A., Harte W.E., Danetz S., Cianci C., Tiley L., Day S., Mullaney D., Yu K.-L., Ouellet C. (1997). Molecular mechanism underlying the action of a novel fusion inhibitor of influenza A virus. J. Virol..

[16] Plotch S.J., O’Hara B., Morin J., Palant O., Larocque J., Bloom J.D., Lang S.A., Digrandi M.J., Bradley M., Nilakantan R. (1999). Inhibition of influenza A virus replication by compounds interfering with the fusogenic function of the viral hemagglutinin. J. Virol..

[17] Yoshimoto J., Kakui M., Iwasaki H., Fujiwara T., Sugimoto H., Hattori N. (1999). Identification of a novel HA conformational change inhibitor of human influenza virus. Arch. Virol..

[18] Vanderlinden E., Goktas F., Cesur Z., Froeyen M., Reed M.L., Russell C.J., Cesur N., Naesens L. (2010). Novel Inhibitors of Influenza Virus Fusion: Structure-Activity Relationship and Interaction with the Viral Hemagglutinin. J. Virol..

[19] Van Dongen M.J.P., Kadam R.U., Juraszek J., Lawson E., Brandenburg B., Schmitz F., Schepens W.B.G., Stoops B., van Diepen H.A., Jongeneelen M. (2019). A small-molecule fusion inhibitor of influenza virus is orally active in mice. Science.

[20] Wang C., Lu L., Na H., Li X., Wang Q., Jiang X., Xu X., Yu F., Zhang T., Li J. (2014). Conjugation of a Nonspecific Antiviral Sapogenin with a Specific HIV Fusion Inhibitor: A Promising Strategy for Discovering New Antiviral Therapeutics. J. Med. Chem..

[21] Xiao S., Tian Z., Wang Y., Si L., Zhang L., Zhou D. (2018). Recent progress in the antiviral activity and mechanism study of pentacyclic triterpenoids and their derivatives. Med. Res. Rev..

[22] Grishko V.V., Galaiko N.V., Tolmacheva I.A., Kucherov I.I., Eremin V.F., Boreko E.I., Savinova O.V., Slepukhin P.A. (2014). Functionalization, cyclization and antiviral activity of A-secotriterpenoids. Eur. J. Med. Chem..

[23] Dang Z., Ho P., Zhu L., Qian K., Lee K.-H., Huang L., Chen C.-H. (2013). New Betulinic Acid Derivatives for Bevirimat-Resistant Human Immunodeficiency Virus Type-1. J. Med. Chem..

[24] Yu F., Wang Q., Zhang Z., Peng Y., Qiu Y., Shi Y., Zheng Y., Xiao S., Wang H., Huang X. (2013). Development of Oleanane-Type Triterpenes as a New Class of HCV Entry Inhibitors. J. Med. Chem..

[25] Yu F., Peng Y., Wang Q., Shi Y., Si L., Wang H., Zheng Y., Lee E., Xiao S., Yu M. (2014). Development of bivalent oleanane-type triterpenes as potent HCV entry inhibitors. Eur. J. Med. Chem..

[26] Song G., Yang S., Zhang W., Cao Y., Wang P., Ding N., Zhang Z., Guo Y., Li Y. (2009). Discovery of the First Series of Small Molecule H5N1 Entry Inhibitors. J. Med. Chem..

[27] Song G., Shen X., Li S., Li Y., Si H., Fan J., Li J., Gao E., Liu S. (2016). Structure-activity relationships of 3-O-beta-chacotriosyl oleanane-type triterpenoids as potential H5N1 entry inhibitors. Eur. J. Med. Chem..

[28] Luo L., Han W., Du J., Yang X., Duan M., Xu C., Zeng Z., Chen W., Chen J. (2018). Chenodeoxycholic Acid from Bile Inhibits Influenza A Virus Replication via Blocking Nuclear Export of Viral Ribonucleoprotein Complexes. Molecules.

[29] Granados A., Petrich A., McGeer A., Gubbay J.B. (2017). Measuring influenza RNA quantity after prolonged storage or multiple freeze/thaw cycles. J. Virol. Methods.

[30] Cai W., Li Y., Chen S., Wang M., Zhang A., Zhou H., Chen H., Jin M. (2015). 14-Deoxy-11,12-dehydroandrographolide exerts anti-influenza A virus activity and inhibits replication of H5N1 virus by restraining nuclear export of viral ribonucleoprotein complexes. Antivir. Res..

[31] Rao X., Huang X., Zhou Z., Lin X. (2013). An improvement of the 2^(-delta delta CT) method for quantitative real-time polymerase chain reaction data analysis. Biostat. Bioinform. Biomath..

[32] Schmittgen T.D., Livak K.J. (2008). Analyzing real-time PCR data by the comparative C-T method. Nat. Protoc..

[33] Basu A., Antanasijevic A., Wang M., Li B., Mills D.M., Ames J.A., Nash P.J., Williams J.D., Peet N.P., Moir D.T. (2014). New Small Molecule Entry Inhibitors Targeting Hemagglutinin-Mediated Influenza A Virus Fusion. J. Virol..

[34] Liao Q., Qian Z., Liu R., An L., Chen X. (2013). Germacrone inhibits early stages of influenza virus infection. Antivir. Res..

[35] Senne D.A., Panigrahy B., Kawaoka Y., Pearson J.E., Suss J., Lipkind M., Kida H., Webster R.G. (1996). Survey of the hemagglutinin (HA) cleavage site sequence of H5 and H7 avian influenza viruses: Amino acid sequence at the HA cleveage site as a marker of pathogenicity potential. Avian Dis..

[36] Ye M., College of Veterinary Medicine, South China Agricultural University, Guangzhou, China (2019). Personal Communication.

[37] Kadam R.U., Wilson I.A. (2017). Structural basis of influenza virus fusion inhibition by the antiviral drug Arbidol. Proc. Natl. Acad. Sci. USA.

[38] Edinger T.O., Pohl M.O., Stertz S. (2014). Entry of influenza A virus: Host factors and antiviral targets. J. Gen. Virol..

[39] Lin D., Luo Y., Yang G., Li F., Xie X., Chen D., He L., Wang J., Ye C., Lu S. (2017). Potent influenza A virus entry inhibitors targeting a conserved region of hemagglutinin. Biochem. Pharmacol..

[40] Russell R.J., Kerry P.S., Stevens D.J., Steinhauer D.A., Martin S.R., Gamblin S.J., Skehel J.J. (2008). Structure of influenza hemagglutinin in complex with an inhibitor of membrane fusion. Proc. Natl. Acad. Sci. USA.

[41] Ekiert D.C., Bhabha G., Elsliger M.-A., Friesen R.H.E., Jongeneelen M., Throsby M., Goudsmit J., Wilson I.A. (2009). Antibody Recognition of a Highly Conserved Influenza Virus Epitope. Science.

[42] Throsby M., van den Brink E., Jongeneelen M., Poon L.L.M., Alard P., Cornelissen L., Bakker A., Cox F., van Deventer E., Guan Y. (2008). Heterosubtypic Neutralizing Monoclonal Antibodies Cross-Protective against H5N1 and H1N1 Recovered from Human IgM(+) Memory B Cells. PLoS ONE.

[43] Si L., Meng K., Tian Z., Sun J., Li H., Zhang Z., Soloveva V., Li H., Fu G., Xia Q. (2018). Triterpenoids manipulate a broad range of virus-host fusion via wrapping the HR2 domain prevalent in viral envelopes. Sci. Adv..

