# Supplementary material for: An Oleanolic Acid Derivative Inhibits Hemagglutinin-Mediated Entry of Influenza A Virus
Source: Viruses. 2020 Feb 18;12(2):225. doi: 10.3390/v12020225 (PMC7077228; doi:10.3390/v12020225)
Supplement: Supplementary file 1 [file viruses-12-00225-s001.pdf]

Supplementary material

1. Chemical structures of oleanane acid (OA-0) and its 11 derivatives

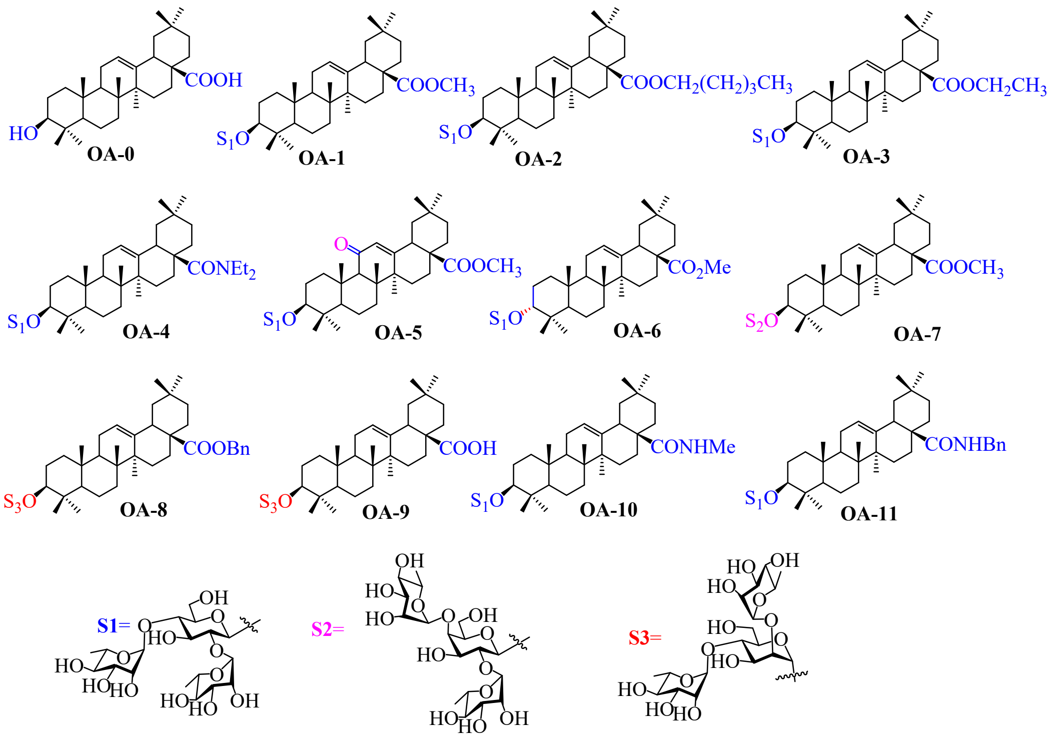

Figure S1. Chemical structures of oleanane acid (OA-0) and its 11 derivatives. OA-8, OA-9, OA-10 and OA-11 were novel synthesized derivatives.

2. Chemical structure identification of 4 new derivatives

Chemical structures of the 4 new derivatives OA-8, OA-9, OA-10 and OA-11 were identified based on data from nuclear magnetic resonance (NMR) and high-resolution electrospray ionization mass spectrometry (HRESIMS) analysis.

OA-8

^{1}H NMR (CD_3OD): δ 7.32-7.37 (m, 5H, Ar-H), 5.32 (t, 1H, J = 3.4 Hz, H-12), 5.08 (s, 2H, Ar-CH_2), 5.06 (d, 1H, J = 2.0 Hz, Rha-H-1), 4.86 (d, 1H, J = 1.4 Hz, Rha-H-1), 4.86 (d, 1H, J = 1.5 Hz, H-1′), 3.97-4.02 (m, 1H), 3.91-3.95 (m, 1H), 3.90 (dd, 1H, J = 8.6, 3.2 Hz), 3.85-3.87 (m, 3H), 3.73-3.79 (m, 3H), 3.68-3.71 (m, 2H), 3.66 (dd, 1H, J = 9.5, 3.4 Hz, Rha-H-3), 3.41 (q, 2H, J = 9.7 Hz), 3.29 (dd, 1H, J = 11.7, 4.0 Hz, H-3), 2.92 (dd, 1H, J = 13.9, 4.3 Hz, H-18), 1.29 (d, 3H, J = 6.2 Hz, Rha-H-6), 1.28 (d, 3H, J = 6.2 Hz, Rha-H-6), 1.15, 1.01, 0.94, 0.94, 0.92, 0.82, 0.59 (each s, each 3H, CH_3); ^{13}C NMR (CD_3OD): δ 177.7 (C-28), 143.5 (C-13), 136.3, 128.1 (two), 127.9 (two), 127.8, 122.5 (C-12), 101.7 (C-1′), 98.9 (Rha-C-1), 93.5 (Rha-C-1), 82.6, 77.2, 76.2, 72.9, 72.6, 72.4, 71.1, 71.0, 70.8, 70.7, 69.5, 69.3, 68.9, 65.8, 60.9, 55.4, 46.7, 45.7, 41.4, 41.3, 39.2, 38.0, 37.9, 36.7, 33.4, 32.5, 32.2, 32.1, 30.2,

28.0, 27.3, 25.0, 23.1, 22.7, 22.6, 21.5, 18.0, 16.6, 16.5, 16.3, 15.7, 14.5; HRESIMS calcd for $C_{55}H_{84}O_{16}Na$ 1023.5652; found 1023.5654.

OA-9

^{1}H NMR (CD_3OD): δ 5.26 (t, 1H, J = 3.4 Hz, H-12), 5.06 (d, 1H, J = 1.7 Hz, Rha-H-1), 4.85 (d, 1H, J = 1.1 Hz, Rha-H-1), 4.84 (d, 1H, J = 1.2 Hz, H-1′), 3.95-4.01 (m, 1H), 3.91-3.95 (m, 1H), 3.90 (dd, 1H, J = 8.6, 3.2 Hz), 3.85-3.87 (m, 3H), 3.74-3.79 (m, 3H), 3.68-3.71 (m, 2H), 3.67 (dd, 1H, J = 9.5, 3.4 Hz, Rha-H-3), 3.41 (q, 2H, J = 9.7 Hz), 3.30 (dd, 1H, J = 11.9, 4.1 Hz, H-3), 2.86 (dd, 1H, J = 13.7, 4.0 Hz, H-18), 1.29 (d, 3H, J = 6.2 Hz, Rha-H-6), 1.28 (d, 3H, J = 6.2 Hz, Rha-H-6), 1.17, 1.02, 0.98, 0.95, 0.92, 0.83, 0.83 (each s, each 3H, CH_3); ^{13}C NMR (CD_3OD): δ 180.6 (C-28), 143.9 (C-13), 122.2 (C-12), 101.7 (C-1′), 98.9 (Rha-C-1), 93.5 (Rha-C-1), 82.7, 77.2, 76.2, 72.8, 72.6, 72.3, 71.1, 71.0, 70.8, 70.7, 69.5, 69.3, 68.9, 65.8, 60.9, 55.5, 46.3, 45.9, 41.5, 41.3, 39.2, 38.0, 37.9, 36.7, 33.5, 32.6, 32.4, 32.2, 30.2, 28.0, 27.4, 25.0, 23.2, 22.7, 22.6, 21.5, 18.0, 16.6, 16.5, 16.4, 15.7, 14.5; HRESIMS calcd for $C_{48}H_{78}O_{16}Na$ 933.5182; found 933.5184.

OA-10

^{1}H NMR (DMSO-d6): δ 5.19 (brs, 1H, H-12), 5.15 (s, 1H, Rha-H-1), 4.67 (s, 1H, Rha-H-1), 4.23 (d, 1H, J = 7.9 Hz, H-1′), 3.76-3.81 (m, 2H), 3.74 (dd, 1H, J = 3.1, 1.1 Hz, Rha-H-2), 3.62 (dd, 1H, J = 3.1, 1.2 Hz, Rha-H-2), 3.57 (d, 1H, J = 11.4 Hz, H-6-1′), 3.49 (dd, 1H, J = 9.5, 2.9 Hz, Rha-H-3), 3.43-3.45 (m, 1H), 3.41 (dd, 1H, J = 9.4, 3.0 Hz, Rha-H-3), 3.36-3.37 (m, 2H), 3.25 (t, 1H, J = 8.5 Hz), 3.11-3.12 (m, 1H), 2.96 (dd, 1H, J = 10.8, 4.8 Hz, H-3), 2.66 (dd, 1H, J = 14.8, 5.6 Hz, H-18), 1.09 (d, 3H, J = 6.1 Hz, Rha-H-6), 1.04 (d, 3H, J = 6.1 Hz, Rha-H-6), 1.03, 0.89, 0.83, 0.81, 0.80, 0.70, 0.59 (each s, each 3H, CH_3); ^{13}C NMR (DMSO-d6): δ 177.3 (C-28), 143.3 (C-13), 121.5 (C-12), 103.3 (C-1′), 100.0 (Rha-C-1), 99.7 (Rha-C-1), 87.9, 76.8, 76.6, 75.6, 74.5, 71.4, 71.3, 70.0, 69.7, 69.6, 69.5, 68.3, 67.8, 59.5, 54.9, 46.7, 45.8, 44.8, 40.6, 39.9, 38.8, 35.7, 33.0, 32.4, 31.9, 31.7, 29.8, 26.9, 26.4, 25.5, 25.2, 22.9, 22.4, 21.9, 17.2, 17.1 (two), 15.8, 15.6, 14.6; HRESIMS calcd for $C_{49}H_{81}O_{15}NNa$ 946.5498; found 946.5505.

OA-11

^{1}H NMR (CD_3OD): δ 7.22-7.71 (m, 5H, Ar-H), 5.38 (d, 1H, J = 1.1 Hz, Rha-H-1), 5.32 (t, 1H, J = 3.3 Hz, H-12), 4.86 (d, 1H, J = 1.1 Hz, Rha-H-1), 4.44 (d, 1H, J = 7.7 Hz, H-1′), 4.40 (dd, 1H, J = 14.7, 5.9 Hz), 4.28 (dd, 1H, J = 14.7, 5.8 Hz), 3.97-4.01 (m, 2H), 3.90-3.95 (m, 1H), 3.85 (dd, 1H, J = 3.3, 1.2 Hz, Rha-H-2), 3.80 (d, 1H, J = 11.9 Hz, H-6-1′), 3.76 (dd, 1H, J = 9.5, 3.3 Hz, Rha-H-3), 3.67 (dd, 1H, J = 12.0, 3.9 Hz, H-6-2′), 3.64 (dd, 1H, J = 9.4, 3.2 Hz, Rha-H-3), 3.60 (t, 1H, J = 8.7 Hz), 3.55 (t, 1H, J = 8.8 Hz), 3.46 (t, 1H, J = 8.0 Hz), 3.39-3.43 (m, 2H), 3.17 (dd, 1H, J = 11.6, 4.0 Hz, H-3), 2.83 (dd, 1H, J = 13.4, 4.0 Hz, H-18), 1.28 (d,

3H, J = 6.2 Hz, Rha-H-6), 1.22 (d, 3H, J = 6.2 Hz, Rha-H-6), 1.17, 1.06, 0.93, 0.93, 0.92, 0.87, 0.61 (each s, each 3H, CH_3); ^{13}C NMR (CD_3OD): δ 178.7 (C-28), 143.8 (C-13), 138.9, 128.0 (two), 127.4 (two), 126.7, 122.7 (C-12), 101.7 (C-1′), 100.6 (Rha-C-1), 100.5 (Rha-C-1), 89.0, 79.0, 77.8, 76.7, 75.0, 72.5, 72.3, 71.0, 70.7, 70.6, 69.4, 68.6, 60.6, 55.9, 46.3, 46.1 (two), 43.0, 42.9, 41.5, 41.3, 39.3, 38.8, 38.7, 36.5, 33.8, 33.0, 32.5, 32.2, 30.2, 27.1 (two), 25.8, 25.1, 23.1, 22.7, 17.9, 16.7, 16.5, 16.4, 15.8; HRESIMS calcd for $C_{55}H_{85}O_{15}NNa$ 1023.2668; found 1023.2652.

3. Antiviral activities of OA-10 against IAV PR8, H9N2 and H3N2 subtypes in A549 cells

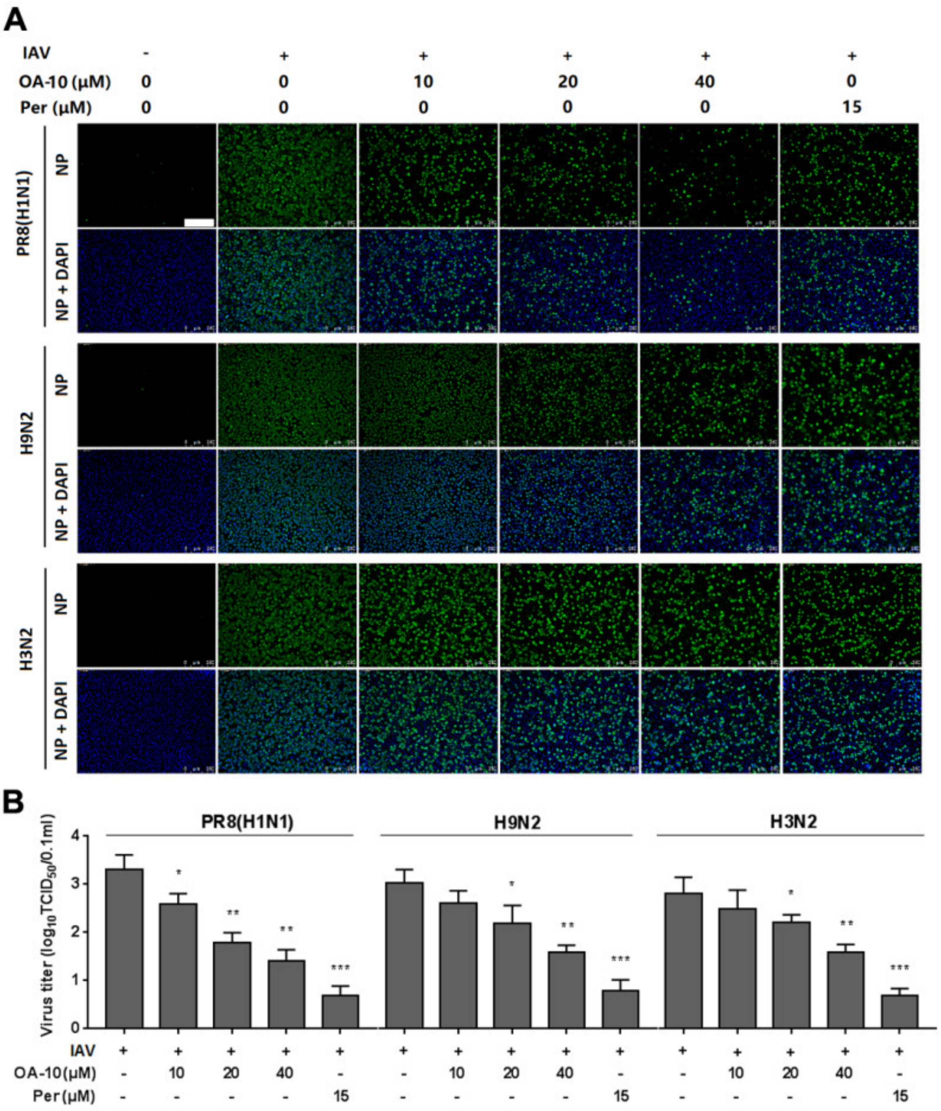

Figure S2. OA-10 inhibited IAV PR8, H9N2 and H3N2 replication in A549 cells. Confluent A549 cells grown in 96-well plates were infected with IAV virus (0.1 MOI) for 1 h at 37℃ and then cultured in fresh medium containing various concentrations of OA-10. (**A**) At 24 hpi, the cells were fixed with paraformaldehyde and the viral NP expression was detected by indirect immunofluorescence assay (IFA). Representative IFA images from three independent experiments were shown. Scale bar: 250 μm. (**B**) At 48 hpi, supernatants of each well were

subjected to viral titer analysis by an end point dilution assay using MDCK cells. *$P < 0.05$, ** $P < 0.01$ and *** $P < 0.001$ compared to the respective virus control.

4. OA-10 does not block IAV binding to A549 cells

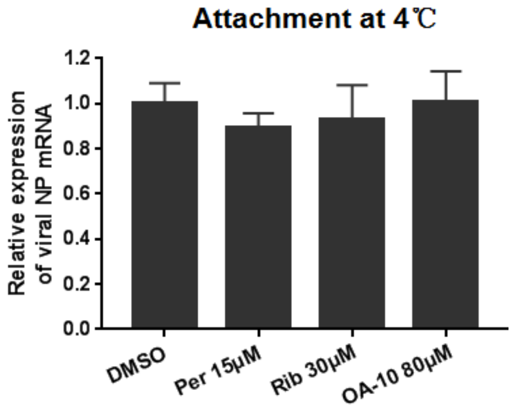

Figure S3. OA-10 does not block IAV binding to A549 cells. A549 cells grown in 24-well plates were infected with 1.0 MOI of H5N1 for 1 h at 4℃ in the presence of OA-10 or control compound (co-treatment). After three washes with PBS, cells in fresh medium were switched to 37℃ for 2 h incubation and harvested at 2 hpi and submitted to NP mRNA analysis by RT-PCR.

5. Amino acid sequence alignment of the full-length HA proteins between strains of A/Vietnam/1203/2004 (H5N1) and A/Duck/Guangdong/99(H5N1)

```
Positions from 1 till 60
A/Vietnam/1203/2004(H5N1)     MEKIVLLFAIVSLVKSDQICIGYHANNSTEQVDTIMEKNVTVTHAQDILEKKHNGKLCDL
A/Duck/Guangdong/99(H5N1)     .......L...N................................................

Positions from 61 till 120
A/Vietnam/1203/2004(H5N1)     DGVKPLILRDCSVAGWLLGNPMCDEFINVPEWSYIVEKANPVNDLCYPGDFNDYEELKHL
A/Duck/Guangdong/99(H5N1)     ........K................................A.......Y..........

Positions from 121 till 180
A/Vietnam/1203/2004(H5N1)     LSRINHFEKIQIIPKSSWSSHEASLGVSSACPYQGKSSFFRNVVWLIKKNSTYPTIKRSY
A/Duck/Guangdong/99(H5N1)     ........................S.........................NI........

Positions from 181 till 240
A/Vietnam/1203/2004(H5N1)     NNTNQEDLLVLWGIHHPNDAAEQTKLYQNPTTYISVGTSTLNQRLVPRIATRSKVNGQSG
A/Duck/Guangdong/99(H5N1)     ...............................................K............

Positions from 241 till 300
A/Vietnam/1203/2004(H5N1)     RMEFFWTILKPNDAINFESNGNFIAPEYAYKIVKKGDSTIMKSELEYGNCNTKCQTPMGA
A/Duck/Guangdong/99(H5N1)     .I....................................A.....................
```

Positions from 361 till 420

```
A/Vietnam/1203/2004(H5N1)    WQGMVDGWYGYHHSNEQGSGYAADKESTQKAIDGVTNKVNSIIDKMNTQFEAVGREFNNL
A/Duck/Guangdong/99(H5N1)    ............................................................
```

Positions from 421 till 480

```
A/Vietnam/1203/2004(H5N1)    ERRIENLNKKMEDGFLDVWTYNAELLVLMENERTLDFHDSNVKNLYDKVRLQLRDNAKEL
A/Duck/Guangdong/99(H5N1)    ............................................................
```

Positions from 481 till 540

```
A/Vietnam/1203/2004(H5N1)    GNGCFEFYHKCDNECMESVRNGTYDYPQYSEEARLKREEISGVKLESIGIYQILSIYSTV
A/Duck/Guangdong/99(H5N1)    ...............................................V.T..........
```

Positions from 541 till 568

```
A/Vietnam/1203/2004(H5N1)    ASSLALAIMVAGLSLWMCSNGSLQCRICI
A/Duck/Guangdong/99(H5N1)    .............................
```

Figure S4. Amino acid sequence alignment of the full-length HA proteins between strains of A/Vietnam/1203/2004 (H5N1) and A/Duck/Guangdong/99(H5N1)

Amino acid sequence identity of the full-length HA proteins between strains of A/Vietnam/1203/2004 (H5N1) and A/Duck/Guangdong/99(H5N1) was calculated as 97.0% using Megalian software. Amino acid sequence of HA2 is presented in red font and different amino acids between two strains are characterized as yellow painting. Only three amino acids are different in HA2 part between the two H5N1 strains.
